# Preferences of Older Immigrants in Nursing Care: A Scoping Review

**DOI:** 10.1093/geroni/igab046.1018

**Published:** 2021-12-17

**Authors:** Viktoria Peters-Nehrenheim, Huerrem Tezcan-Guentekin, Martina Roes

**Affiliations:** 1 DZNE, German Center for Neurodegenerative Diseases, Nordrhein-Westfalen, Germany; 2 ASH, Berlin, Berlin, Germany; 3 German Center for Neurodegenerative Diseases (DZNE), Witten, Nordrhein-Westfalen, Germany

## Abstract

Background: Worldwide, our societies are characterized by an increasing diversity, greatly contributed by immigrants. When in need of care older immigrants face various barriers and serious challenges in terms of unmet preferences. To provide person-centered care, health care professionals need to consider the personal background of immigrants to identify and assess their individual preferences. Objective: To understand how preferences of older immigrants in nursing care are defined and how they can be assessed. Methods: A scoping review will be conducted to identify and analyze preferences of older immigrants across health care settings. Preliminary results: The literature search revealed that older immigrants in need of care define their preferences in terms of expectations and priorities. Differences among immigrants are related to the age of the person at the time of immigration, on cultural differences and/or on how the concepts of preferences in the country of origin is understood.

